# “The Light at the End of the Tunnel”: Experiences of LGBTQ+ Adults in Portuguese Healthcare

**DOI:** 10.3390/healthcare10010146

**Published:** 2022-01-13

**Authors:** Mara Pieri, Joana Brilhante

**Affiliations:** Centre for Social Studies, University of Coimbra, 3000-995 Coimbra, Portugal

**Keywords:** LGBTQ+, visibility, discrimination, Portugal, healthcare

## Abstract

This work analyses experiences of LGBTQ+ people accessing healthcare in Portugal. A total of 32 semi-structured interviews were conducted with queer adults (18–59 years old). The thematic analysis and thematic networks brought to light how structural cis-heteronorms are compliant with the maintenance of invisibility regarding sexual and gender diversity. As a consequence, experiences of direct and indirect discrimination show us how crucial it is to have well prepared healthcare providers, capable of embracing diversity and creating safe spaces that allow us to shorten the path between Portugal’s progressive legal frame and the people lived experiences.

## 1. Introduction

The importance of LGBTQ+ (lesbian, gay, bisexual, trans, queer and others) issues in healthcare has grown in the last two decades as a reflection of expanding awareness about the need to address the multiple discriminations connected to sexual and gender diversity in public and private spaces. This article analyses the narratives of LGBTQ+ people regarding their experiences in the Portuguese healthcare system. The objective is to understand what are the lived experiences of LGBTQ+ people. Additionally, the article aims at analysing whether such experiences reflect the Portuguese (progressive) legal framework and providing insights to improve further intervention.

Recent research shows that access to healthcare and adequate treatments is still reduced for LGBTQ+ people of all ages [1,2,3]. Although the differences between healthcare systems and the inner diversity within the LGBTQ+ spectrum can point to a variety of situations, three elements are consistently present: compulsory cis-heteronormativity, invisibility, and insufficient knowledge of healthcare professionals.

Heteronormativity is a concept derived from the definition of “compulsory heterosexuality” coined by Adrienne Rich [4]: in its original meaning, it referred to the societal expectation that heterosexuality is the only desirable choice and to the social roles that are moulded on such expectation. Queer studies expanded the notion to “compulsory heteronormativity” [5] to indicate the pervasive norm that endlessly reproduces a linear correspondence between sex, gender, and sexual orientation. Heteronormativity is so pervasive and rooted in contemporary societies that it becomes considered a natural aspect of human life and societal organisation. The overlapping between nature and western culture and the constitution of reality as a list of binary opposites is still today constitutive of health and biomedicine [1], as it is evident in the persistent use of oppositions such as masculine/feminine, men/women, heterosexual/homosexual, normal/deviant, natural/abnormal. In particular, the binary contrast between male and female retains crucial importance in defining the ways bodies are subjected to biomedical scrutiny and experiences of illness and pain are defined [3]. In the relationships between patients and healthcare providers (henceforth HCP), compulsory heteronormativity can translate into the expectations that patients show an alignment in terms of sex, gender, sexual orientation and even sexual behaviours or reproductive trajectories. Indeed, “when medical authorities define male-men and female-women as necessarily distinct beings who operate as halves or sides of a human whole, their efforts encourage and naturalize heterosexuality at the expense of other forms of sexual expression, desire, connection, and practice” [2].

Compulsory heteronormativity also assumes the form of an invisible, though persistent, pressure to conform, which can have serious consequences on the trajectories of LGBTQ+ people in healthcare: they can be treated differently, discriminated against (directly or indirectly) or even be victims of violence and aggression. In a report compiled in 2015, ILGA-Portugal made a survey (to know more about ILGA’s 2015 survey: https://ilga-portugal.pt/ficheiros/pdfs/igualdadenasaude.pdf (accessed on 2 November 2021)) directed to LGBQT+ people around their experiences of visibility in healthcare access. A large percentage of the sample (83%) responded they had already encountered HCP that had assumed they were heterosexual and/or cisgender. Additionally, 29% of them declared they had never disclosed their sexual orientation and/or gender identity to a healthcare provider [6].

This leads us to a second dire obstacle for LGBTQ+ people in healthcare systems: visibility. Coming out as LGBTQ+ to healthcare professionals can be a risky choice. The fear of discrimination is constantly at play, as patients worry that they will be treated with less care or be victims of prejudice [7,8]. As it happens in other contexts, such as workplaces and families, each individual tends to make choices of visibility based on strategic evaluations of the benefits but, mostly, of the potential threats that could be associated with it [9]. Coming out is a performative act to be repeated hundreds of times in a lifetime and the cumulative stress of such evaluations contributes to maintaining one’s sexual orientation or gender identity secret unless strictly necessary. However, invisibility also determines costs and risks. Like other marginalised groups, LGBTQ+ people tend to avoid regular access to healthcare and recur to treatments only in serious situations. Moreover, they show reduced involvement in screening programs and routine check-ups. As a consequence, LGBTQ+ people are more likely to receive a late diagnosis and show higher rates of comorbidity [3]. The invisibility of LGBTQ+ people in healthcare contexts is exacerbated by a more general absence of sexuality as a relevant matter in health and as an important aspect of body-mind wellness. In this regard, the only areas in which sexual orientation and gender identity are consistently considered as relevant variables are gender-affirmation surgeries, treatment of STDs or HIV/AIDS, and intervention on intersex children. However, considering LGBTQ+ people as patients with specificities and needs that go beyond these categories and as holders of the right to be treated equally is a beneficiary step for healthcare systems as a whole [10].

A third important obstacle to be mentioned is the scarcity of curricula in healthcare careers directed at LGBTQ+-related issues. In Portugal, as it happens in many other European countries, medical doctors, nurses and other HCP can conclude their formative curricula without ever coming in contact with resources related to LGBTQ+ issues or without receiving even basic training on that matter [11,12]. The lack of specific knowledge, together with the social bias to which every HCP is exposed, constitute a major factor of reproduction of compulsory cis-heteronormativity, invisibility and, ultimately, discrimination.

Following a considerable wave of legislative change that placed the country amongst the five most LGBTQ+-friendly in Europe [13], the Portuguese government recently approved an important resolution to repair the absence of a proper framework for LGBTQ+ patients in healthcare. The “National Health Strategy for LGBTI people” [14] provides guidelines for advancing literacy on LGBTI issues amongst HCP. It also encourages measures to develop best practices for the access of LGBTQ+ people in the healthcare system.

In this article, we analyse the narratives of LGBTQ+ people regarding their experiences in the Portuguese healthcare system: the focal issues provided by the literature discussed guide the analyses. After a brief overview of the methodological aspects, we first discuss examples that reveal the multiple layers of discrimination and the lack of knowledge on LGBTQ+ issues amongst HCP. Secondarily, we focus on invisibility and its relation with the maintenance of a system that keeps sexual and gender diversity marginalised. As we highlight in the discussion of the results, the main goal of the analysis is understanding to what extent the narratives collected are in line with the Portuguese (progressive) legal framework. Thanks to such analyses, we aim at providing insights to improve further intervention.

## 2. Material and Methods

### 2.1. Procedure

This paper assembles data from the interviews of two qualitative studies conducted with Portuguese LGBTQ+ individuals between 2017 and 2021. Both investigations result from the doctoral projects of the authors in which the lived experiences of the participants were the main focus. The prevalence of statements regarding interactions with HCP and the healthcare system led to a careful analysis of these discourses that will be reported in this paper.

The recruitment for participants was made with the use of different methodologies: online forums, contact with associations, a call shared on social media, newsletters, conversations with LGBTQ+ activists that served as gatekeepers [15] and through snowball sampling [16].

Due to COVID-19 world pandemics, necessary adjustments had to be made to data collection. This resulted in the adaptation of part of the interview process to online format using Zoom platform.

A total of 32 semi-structured interviews [17,18] were considered, all conducted in Portuguese and with an average length of 1h30. This interview methodology is well explored in social health studies [19,20,21].

All participants took part to the study on a voluntary basis and agreed with an informed consent that allowed the use of the data and audio recording. All interviews were anonymised: the names used in this work are fictional and the age of participants is presented in a 10-year range.

Recordings were transcribed verbatim using MAXQDA software and the interpretation of data employs thematic analysis [22,23] and thematic networks [24].

### 2.2. Participant’s Information

Regarding sexual orientation people identified as being lesbian (6), gay (9), bisexual (4), pansexual (3), asexual (1) and demi-sexual (1). Whereas gender identity, identifications were Trans (2 men, 1 woman), cisgender (8 women, 3 men), non-binary (2) and gender fluid (1). Some people identified with more than one category, and in some cases people did not mention their sexual orientation nor gender identity directly. Two people identified also as non-monogamous.

All 32 participants are Portuguese or living in Portugal; they come from different places in the country–North, Centre, South and both autonomous regions, the archipelagos of Azores and Madeira.

Concerning the age of participants, Table 1 gathers the information divided into 5 major groups.

A substantial part of the interviewees (13) had one or more chronic illnesses which interfere directly with their everyday activities. Some of the chronic illnesses included are: cholinergic urticaria; rheumatoid arthritis; fibromyalgia; cancer-related conditions and adrenal insufficiency. Additionally, references to mental health issues were conspicuous during data analysis, mostly related to depression and/or anxiety.

### 2.3. Research Design

The analysis of data was made through the use of thematic analysis and thematic networks. Firstly, we started with simple coding, mainly descriptive. Afterwards, we clustered the codes within broader categories, and, finally, the categories within overarching themes (health and care), that we united in just one comprehensive theme–healthcare.

The inductive process led us to the relevance of healthcare, as the theme gathers 162 segments of coded text, with rich and meaningful statements. Interestingly, the codes from healthcare categories showed even in interviews where we didn’t foresee it, as the cases of healthy, cisgender participants.

**Figure 1:**
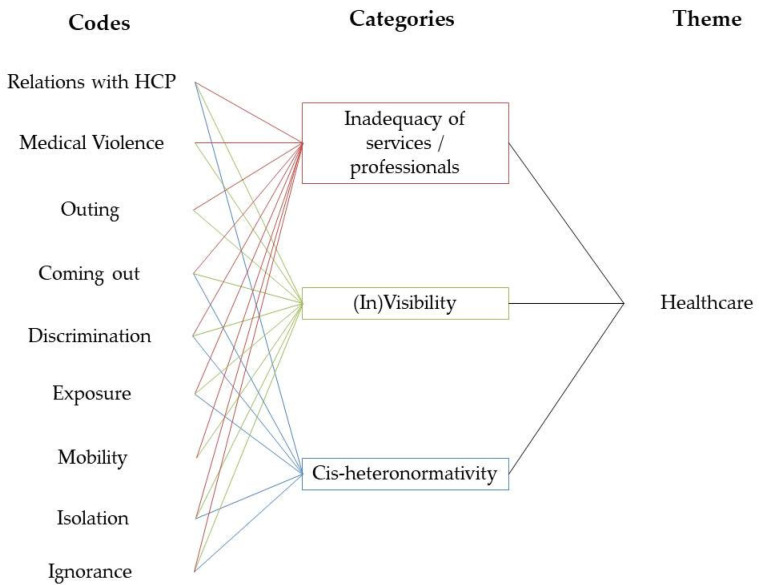
Thematic network of data analysis.

The thematic network (Figure 1) illustrates the analytic process as follows:

The thematic network is a useful tool to organise a thematic analysis [24] and the structure of this paper was built accordingly. The results presentation and the discussion are centred in the three main categories that emerged from data analysis: inadequacy of healthcare services and/or professionals, LGBTQ+ visibility and cis-heteronormativity. As all categories are directly or indirectly related, we divided the presentation of the results into two main sections: “Heteronormativity and prejudices” and “Shedding light over LGBTQ+ (in)visibility”.

## 3. Results

### 3.1. Heteronormativity and Prejudices

Numerous narratives reflect the compulsory heteronormative matrix of the Portuguese healthcare system. One of the ways it manifests is the systematic erasure of choices and experiences that do not fall into the expected alignment, such as monogamous heterosexuality. Jasmim, a polyamorous non-binary interviewee, says:

*“If I go to the gynaecologist [...] or an appointment for STDs, to do the routine test, the questions are not made for me. Also, they ask how many persons I had sex with in the last six months, that’s ridiculous! I can tell how many risky relationships I had but I’m not supposed to tell how many partners I was with!”*

Jasmim, 30–39

The excerpt shows how even in contexts in which sexuality is at the centre of the discussion, it is presumed to be a heterosexual, monogamous, and procreative one. The lack of options to signal relations and behaviours that diverge from the assumed “normalcy” creates the sense of being invisible, hence, of not existing.

The heteronormative organisation of the healthcare system is so embedded into the very structure of cultural reproduction that it even resists legislative changes. In the case of Portugal, despite recent measures to raise awareness about the importance of LGBTQ+ patients and changes in the regulations, many cases report a mismatch between legislation and practices. The case of Graça, an interviewee who has a relationship with another woman and had a baby with her partner, is paradigmatic. While she was pregnant, they had some encounters with the doctors. During one of these check-ups, the doctor told her that on the day of the birth her partner should proceed to adopt the baby:

*“The doctor suddenly told me “I was looking into it with the person that deals with papers and stuff, she knows about your case, I did my research and when the time comes everything will be easy, you’ll just have to proceed with an adoption”. And you know when you get shocked? You’re not expecting this...I wanted to say something at the moment but I couldn’t say anything, I just kept thinking...an adoption? I went home and said, “When the time comes we’ll see what happens”.*

Graça, 20–29

The doctor gave Graça and the whole staff completely wrong information, that did not take into account the legislative developments promulgated in Portugal. According to the Law approved in 2016 (Lei n. 17/2016), same-sex couples do not have to proceed with adoption when one of the two partners gives birth: their children are to be registered with the same procedure used for heterosexual couples. The mistake did not involve only the doctor but the whole unit that accompanied the birth of Graça’s child: nurses, administrative staff, and assistants advanced with a process of adoption without any question. After realising they had been forced to undergo an unnecessary bureaucratic procedure, Graça and her partner took months to repair the error. The adoption was finally overturned to regular registration after the intervention of a lawyer.

The example suggests two interesting elements for analysis. In the first place, Graça and her partner were amongst the very first lesbian couple to give birth in that hospital. Indeed, in other moments of the interview, she recalls how they were objects of major curiosity and even excessive attention from the HCP during the hospitalisation. HCP were not prepared to deal with a homosexual couple giving birth, on a procedural level nor behavioural level, although four years had passed between the promulgation of the law on same-sex parenting and that moment. In the second place, the story shows the profound asymmetry of power existing not only between the doctor and the patients but also between the doctor and the other HCP of the unit. In such a regime of asymmetry, the knowledge transmitted by the doctor goes unchecked and is assumed to be rightful a priori.

The lack of knowledge on LGBTQ+ issues by HCP is a reason for concern that emerges in other interviews as well. In some cases, it becomes visible during the encounter between patients and HCP. In other cases, it is expressed as a preventive worry when it comes to providing support to LGBTQ+ people in need of medical support, especially in small centres and in the islands:

*“In the islands, we have this issue, I cannot recommend a doctor for a trans person. Last week a colleague called me, she needed a recommendation [for a trans person] [...] I was flustered, scared, because the person was expelled from home, in a state of emotional fragility: we need to be very careful. My colleague said: “We have to be careful, what doctor can he see? If the doctor is not willing to make it work, the guy will be destroyed. [...] It must be a doctor with an open mind, not one of those that would say “Take a pill and go home, you’re just depressed”. But I didn’t know anyone.”*

Emanuel, 20–29

The situation told by Emanuel is echoed in the narratives of activists involved in making efforts to create effective support networks. The issue is particularly important for what concerns trans and non-binary young people, who are more likely to be exposed to medical violence as well as other forms of discrimination by families and workplaces [12]. In this regard, finding an HCP prepared to deal with trans and non-binary persons is crucial to guarantee that an already heavily medicalised process can proceed safely and respectfully. Several interviewees report being sent to see psychologists and psychiatrists as the first entrance to healthcare. Although the incidence of mental health issues amongst the LGBTQ+ population cannot be overlooked [25,26], the narratives raise concern that the lack of staff trained to deal with sexual and gender diversity may bring excessive attention to the psychological level and disregard the other elements of health and wellbeing.

Several interviewees report experiences of direct discrimination and stigmatisation. The next two excerpts refer to different contexts: the first happened to Jorge, one of the interviewees, during a blood donation; the second to Julia, during a routine check for chronic pain:

*“The nurse started to ask if I had had a sexual relationship, the usual. And I answered as always. She asked if it was with my girlfriend and I answered: “No, with my boyfriend”. So she stood up and went to ask the doctor whether homosexuals could donate blood and the doctor said they couldn’t. The doctor came to me…[…]I felt furious. […] The most ridiculous thing the doctor said was: “A homosexual man cannot give blood because anal sex causes a higher risk of contagion with HIV”. And this is just completely stupid”.*

Jorge, 20–29

*“I was at the doctor for a routine check. She asked me if I used condoms and I said: “No, I don’t need to, I have a girlfriend”. She knows about my illness, fibromyalgia…and she asked me whether the fact of being lesbian had to do with fibromyalgia! […] She said my illness could have something to do with the fact that I had to hide it to many people…and maybe on an emotional level it had had an impact”.*

Julia, 30–39

In both cases, interviewees unveil the texture of homophobic prejudices that still permeate healthcare contexts. In the first example, both the nurse and the doctor reproduce an old stereotype according to which gay people cannot donate blood because their sexual practices are exposed to infections. In this regard, it is worth mentioning that donation by gay men is allowed by Portuguese legislation since 2016. Again, at the time of the interview, four years had passed since the legal implementation but there seems to be a mismatch between the formal level and the practices enacted.

In the second case, the prejudice comes in a more subtle form, which links an illness with the potentially disruptive effects of invisibility as a lesbian. The treatment of chronic pain in women is often subject to gendered bias, according to which HCP tend to discredit the gravity of symptoms or disbelieve patients’ accounts [27]. Although the emotional component of chronic pain is demonstrated [28], there is no scientific basis to explain a chronic illness as a direct expression of invisibility.

In both examples, the voice of the HCPs comes embedded into a sense of entitlement and power asymmetry compared to the patient, which makes it difficult for Jorge and Julia to respond to the blatant misleading information in any way.

### 3.2. Shedding Light over LGBTQ+ (in)visibility

The importance of having well-prepared HCP regarding LGBTQ+ specificities is becoming fairly explored in academic work [20]. Health disparities of queer individuals have been identified when in comparison with cis-heterosexual people, and the disclosure of sexual orientation and/or gender identity is associated with more positive results in care [8]. However, visibility is by no means easy: as the previous section showed, it can lead to direct discrimination, stressful situations, and suffering. Using the words of Eliason and Schope “disclosure can be dangerous, and safety is an important consideration in disclosure” [29].

In this section, we will use examples that emerged during the interviews, to contribute to the understanding of LGBTQ+ (in)visibility in healthcare. In several of our interviews, people often refer to feeling as if they did not “belong”. The first excerpt by Jasmim, discussed in the previous section, is an example of it; others refer to HCP automatically assuming they need contraception if they are read as cis-women; in the case of Graça, her partner was given a hospital badge saying “father” when she was to enter the labour room. These unintentional, though consistent, forms of exclusion contribute to feelings of inappropriateness, shaping places and spaces that are groomed to be used by people that correspond to the expectations of the hetero-norm and marginalising diversity. To exist, diversity has to be affirmed or assumed, reclaiming a space that by default is not prepared to embrace it.

It is no new subject that (in)visibility is a key aspect regarding LGBTQ+ identities [30] and the decision between being visible or invisible is, many times, a negotiation that takes into account the sense of vulnerability or risk. Invisibility is fed both by the normative system (institutions and professionals) and LGBTQ+ people, who for several reasons choose to go unseen. It was the case of Lisa, an interviewee with a long story of chronic pain. When she finally met an HCP that believed her pain was real, she preferred not to come out as pansexual for fear that it would change the relationship with the doctor:

*“In the beginning doctors didn’t believe in my pain. So… but when I finally found a doctor that took my situation seriously, who is a specialist in people with chronic pain, he asked me if I had an active sexual life. Because it was a good thing, because I need to do back exercise.“*

Lisa, 20–29y

A different situation was described by Zé, an LGBTQ+ activist. He spoke about an event where a young boy came to him searching for help as his boyfriend found out he was HIV positive. Zé said that he advised the boy to get tested too, but he strongly refused. Zé thus decided to go to the local health centre to see if he could do something to help in the situation. He described the situation as follows:

*“It was the people at the health centre that spoke to me, in public, about the case, and there I understood the reason why that boy, that teenager, didn’t want to get [HIV] tested or anything like that.”*

Zé, 40–49y

During his visit to the health centre, HCP gave Zé all the information about the boy and other private aspects of his case. Zé was shocked about the lack of privacy and said “these people (HCP) speak [publicly] about everyone’s illnesses”.

It is not the aim of this work to discuss the major importance of professional secrecy, but it must not go unmentioned its relevance in healthcare, in particular for a social group that is often swamped with the fear of outing. Outing describes a situation where the sexual orientation of someone is revealed against their will [31]. It is particularly relevant when analysing data from small, peripheral areas when anonymity is difficult or even impossible, given the networks of relations that make everyone known to everyone. Specially in this kind of setting, HCP must be sensitive in providing safe places and honour their patients’ privacy.

This example is particularly significant also for another element. The boy who found out his boyfriend was HIV positive did not feel safe to access the healthcare system and seek for help there: on the contrary, he felt safer in asking for support from an LGBTQ+ activist. The story shows the importance of activists as mediators in creating safe corridors of access to healthcare [32].

As Zé continued the story, he said that the boy’s boyfriend was so discriminated against by locals that he went to live abroad. Concerning the boy who sought help, he started to have “psychological problems”, in Zé’s words, and dropped out of school.

Zé’s statement is of crucial importance. It triggers important reflections on (in)visibility from many perspectives. In the first place, it shows the invisibility experienced by LGBTQ+ people through isolation and marginalisation. Moreover, it refers to the invisibility that pushes people to migrate from their own country after discrimination. Additionally, it provides a taste of how rapid it can be the change from invisibility to overexposure and hyper-visibility, and how harsh the consequences of such change can be. Finally, it unveils the relations between all these aspects and HCP within healthcare systems.

Another interesting insight comes from Marta, married to another woman for more than 7 years at the time of the interview.

*“The worst part of my sister not accepting [my sexual orientation] was the fact that my niece, that is a [healthcare provider], also stopped speaking to me. (…)When she [sister] thinks like that I think “well… she is in her 60 s, poor wretch, let her be”. But a [healthcare provider], in her 30 s thinking the same way… to me… well, it hurts a lot”.*

Marta, 50–59y

Marta expected her niece to be at ease with her coming out not only as a young person but even more as an HCP. Her account reminds us that HCP are also people, have families, friends, and colleagues. The expectation about their behaviour, when they are at work and even outside workplaces, is that they are open to LGBTQ+ issues. As significant social actors, this is an important issue to keep in mind.

The decisive importance of good HCP, that are well informed and capable of creating secure places for LGBTQ+ people to disclose in safety, becomes clear in Margarida’s testimonial. As a trans woman from a small peripheral area, she spoke about her poor experience with her first psychologist, that suggested she was a gay man. She related how she felt alone, without references or help: the situation affected her to the point of dropping out of school and causing much psychological distress. She said:

*“It was alone, because here [where she lives] I am the only one who transitioned from boy to girl. So I was alone on this path. I searched for a psychiatrist Dr. [name of the doctor] and then yes. She was the one that helped me a lot and redirected me to [name of the city]. And it was where they diagnosed me as such. So there it was… there I really found the light at the end of the tunnel”.*

Margarida, 30–39y

Although finding that doctor was life-changing for Margarida, it was not just in the metaphorical sense. To be able to get all the medical help she required, Margarida had to live in a big Portuguese city, far away from her home, work, family and friends for one year. The concentration of specialised aid for trans people in the big urban cities is also a factor that contributes to the maintenance of the disparities regarding access to healthcare. The inadequacy of knowledge about trans issues in the HCP working in the small, peripheral area where Margarida lives, exacerbates her isolation and generates psychological costs. At the same time, the system of invisibility reproduces itself so that ignorance and lack of knowledge about gender diversity remain unvaried.

## 4. Discussion and Conclusions

The results discussed demonstrate a general adherence to the literature discussed in showing that LGBTQ+ people encounter several obstacles in their access to healthcare. The presence of cis-heteronormative patterns, the awareness about prejudices and discrimination or the fear of suffering consequences on the treatment received are all factors that put LGBTQ+ people in a complex web of risks and strategies whenever they have contact with an HCP [1,3].

The examples show a great variety of factors at play in the interplay between LGBTQ+ patients and HCPs.

In the first place, LGBTQ+ people experience disadvantages and difficulties before accessing healthcare services. With some of the interviewees, the reluctance to recur to check-ups and exams is justified by the suffering of previous experiences of discrimination; in other cases, it is triggered by a preventive concern, or even as avoidance of social exposure. These results go in line with the previously mentioned report made by ILGA-Portugal (2015) [6], where the authors connected the reluctance of accessing healthcare services with the fear of discrimination and invisibility. In our study, the fear of social exposure represents an additional important element. This particular concern seems to be of major importance when analysing data from smaller, peripheral settings.

The heteronormative matrix that construes healthcare contexts is perceived as a deterrent to visibility. By not making space for the possibilities outside the cis-heteronorm, those existences are erased and made invalid, contributing to the perpetuation of marginalisation and stigma. As previous works also demonstrate [3,8] the costs of late check-ups, discrimination, and lack of connection with HCPs augment the already existing fragility of LGBTQ+ populations and exacerbates forms of inequality created by social marginalisation. Additionally, and in line with the recent work of Henriquez and Ahmad (2021) [33], our study also emphasises how LGBTQ+ people, especially those living in small centres, peripheries, and in the islands, are particularly touched by such multiple and intersectional vulnerabilities, especially relevant for trans, non-binary and young LGBTQ+ people.

In the second place, the experiences of LGBTQ+ people once they access healthcare systems are also marked by multiple levels of vulnerability. Although visibility is encouraged as an important step to contribute to inclusivity and representation, even in healthcare [2,6], the interviews collected demonstrate the costs and the risks it implies. As the stories discussed show, the choice of coming out to HCPs is always the result of a process of negotiation between expectations, fears, social pressures and the signals of inclusion provided by the context. This process often leads LGBTQ+ patients to prefer invisibility over visibility, even when mentioning their sexual orientation and/or gender identity would be important for the clinical aspects (as in the case of STSs check-ups, for example). Invisibility, thus, guarantees the maintenance of integrity and the sense of safety, which is a crucial part of the experience as patients.

Finally, the direct or indirect discrimination experienced by interviewees happens in contexts in which specific measures to boost inclusion in healthcare and legislative framework are in place to provide formal protection against any form of mistreatment. The mismatch between the formal legislative framework and the actual practices is a fundamental aspect to reflect upon. If it shows that laws are not enough, per se, as Davy and Siriwardena [10] note, it also suggests that the ways laws are received, enacted, and respected is fundamental to creating inclusive and safe healthcare systems.

Given this context, data collected showed the need to invest in multiple aspects to improve the access to healthcare for LGBTQ+ people.

As already highlighted, healthcare spaces need to be ready to promote a culture of inclusion and respect. This culture needs to be built through the consistent, capillary, and specific education of HCP about LGBTQ+ lives, starting with the basic curricula. Without measures that specifically tackle the preparation of HCP on LGBTQ+ issues, healthcare systems are safe for some, and profoundly discriminatory for others [34]. Which leaves the principle of equal healthcare access to everyone failing to comply. Oriented preparation for HCPs can also represent a valid element to bring different balance in the power asymmetries that emerged in the interviews.

Moreover, the results of the research point to the importance of investing in safety: LGBTQ+ patients need to rely on practices that will not deny them the right to privacy or let them be driven to invisibility.

The study is inevitably informed by shortcomings and possibilities of further improvement. The onset of the COVID-19 pandemic during the final phase of the data collection forced methodological changes: some interviews scheduled to be in presence had to be done online and thus are informed by a different dynamic in the engagement with the process. At the time, the pandemic was just at the beginning and had not assumed the long-term form it has today. Therefore, data do not reflect the consequences it certainly had in the life of participants in the following months. Future investigations are needed to reflect on what changed during the pandemic and how it particularly affected access to healthcare for Portuguese LGBTQ+ people. Moreover, a larger sample and other questions could have incited a deeper reflection on further aspects of the experience with healthcare system, such as, for example, the difference between access to private and public facilities, differences amongst age cohorts, class or education.

Healthcare systems and HCP hold a crucial role in the fight against homo-transphobic discrimination. As the stories discussed in this article confirm, they are fundamental social actors and need to be principal interlocutors of LGBTQ+ politics. They can also provide a solid contribution in transforming laws into practices, becoming reference points for activists and, if needed, being “the light[s] at the end of the tunnel” for LGBTQ+ patients.

## Figures and Tables

**Table 1:** Participants ages in a 10-year range.

Age	Participants (n)
<20	2
20–29	9
30–39	16
40–49	1
50–59	4
Total	32

## Data Availability

Not applicable.
